# DNA Methylation at the DAT Promoter and Risk for Psychopathology: Intergenerational Transmission between School-Age Youths and Their Parents in a Community Sample

**DOI:** 10.3389/fpsyt.2017.00303

**Published:** 2018-01-10

**Authors:** Silvia Cimino, Luca Cerniglia, Giulia Ballarotto, Eleonora Marzilli, Esterina Pascale, Claudio D’Addario, Walter Adriani, Renata Tambelli

**Affiliations:** ^1^Department of Dynamic and Clinical Psychology, Sapienza University of Rome, Rome, Italy; ^2^Faculty of Psychology, International Telematic University Uninettuno, Rome, Italy; ^3^Department of Medical Surgical Sciences and Biotechnology, Sapienza University of Rome, Rome, Italy; ^4^Faculty of Bioscience and Technology for Food, Agriculture and Environment, University of Teramo, Teramo, Italy; ^5^Department of Clinical Neuroscience, Center for Molecular Medicine, Karolinska Institutet, Stockholm, Sweden; ^6^Center for Behavioral Sciences and Mental Health, Istituto Superiore di Sanità, Rome, Italy

**Keywords:** epigenetics, DAT, 5′-untranslated region, variable number of tandem repeats polymorphism, genotype, methylation, psychopathological symptoms, intergenerational transmission

## Abstract

**Background:**

The effect of gene polymorphisms and promoter methylation, associated with maladaptive developmental outcomes, vary depending on environmental factors (e.g., parental psychopathology). Most studies have focused on 0- to 5-year-old children, adolescents, or adults, whereas there is dearth of research on school-age youths and pre-adolescents.

**Methods:**

In a sample of 21 families recruited at schools, we addressed parents’ psychopathological symptoms (through SCL-90-R); offspring emotional–behavioral functioning (through CBCL-6–18); dopamine transporter gene (DAT1) for epigenetic status of the 5′-untranslated region (UTR) and for genotype, i.e., variable number of tandem repeats polymorphism at the 3′-UTR. Possible associations were explored between bio-genetic and psychological characteristics within the same individual and between triplets of children, mothers, and fathers.

**Results:**

DAT methylation of CpG at positions M1, M6, and M7 in mothers was correlated with maternal (phobic) anxiety, whereas in fathers’ position M6 was related to paternal depression, anxiety, hostility, psychoticism, and higher Global Severity Index (GSI). No significant correlations were found between maternal and offspring DAT methylation. Significant correlations were found between fathers’ methylation at CpG M1 and children’s methylation at CpG M6. Linear regressions showed that mothers and fathers’ GSI predicted children’s methylation at CpG sites M2, M3, and M6, whereas fathers’ GSI predicted children’s methylation at CpG sites, particularly M1, M2, and M6. Moreover, offspring methylation of DAT at CpG M2 predicted somatic complaint, internalizing and attention problems; methylation of DAT at CpG M6 predicted withdraw.

**Conclusion:**

This study may have important clinical implication for the prevention and treatment of emotional–behavioral difficulties in children, as it adds to previous knowledge about the role of genetic and environmental factors in predicting psychopathological symptoms within non-clinical populations.

## Introduction

In the last two decades, emerging evidence from the gene–environment (GxE) interaction literature has allowed an increased knowledge about underpinning mechanisms responsible for the onset and maintaining of psychological difficulties in childhood (1). In particular, in accordance with the Developmental Psychopathology theoretical framework (2), psychopathological risk has been defined as the result of a complex and dynamic interplay between genetic and environmental risk factors; this perspective is known as a “multiple levels of analysis” approach (3, 4). Recent research provided evidence that epigenetics may serve as possible pathways through which relational experiences interact with genes and produce (and/or sustain) changes in behavior. It has been proposed that epigenetic alterations are biological responses to environmental input. Moreover, these modifications seem to have a role in the onset and maintaining of psychopathology (5, 6). Among environmental risk factors, several studies have shown that parents’ psychopathological problems are crucial in affecting children’s mental health (7), fostering the onset of both internalizing and externalizing problems (8, 9). Furthermore, a life-long stability of these psychopathological symptoms in children has also been evidenced (10, 11), which in turn would contribute to further intergenerational transmission of psychopathological risk throughout the life span. The biological factors underlying this kind of transmission have been suggested to be related with both epigenetic modifications (i.e., histone acetylation or DNA methylation) and genotype vulnerability (i.e., allelic polymorphism). In particular, a large body of research has evidenced that human behavior and mood are regulated primary by various neurotransmitters, particularly serotonin and dopamine, which act at synapses by stimulating or inhibiting the flow of an impulse between neurons in the crucial limbic and/or cortical brain areas (12, 13). Consequently, a dysregulated neurotransmitter activity may stem from altered release or reuptake; this may be due to changes in function or expression of key proteins, which has been associated, in turn, with (epi)genetic variability at both levels of allelic genotype and of acetylation/methylation (14, 15). This, nowadays, represents a central process to understand alterations of affective–behavioral functioning.

Genetic polymorphism has been defined as the occurrence of variation in a gene, resulting in the presence of two or more alleles at one locus in a determined population, where the minimum frequency of the least common allele is 1% or more (16). Epigenetic mechanisms refer to any process affecting the regulation and expression of genetic functions and ultimately modulating the protein levels in the brain, without alterations in the DNA sequence (17). Generally, DNA methylation occurs in cytosines that are adjacent to guanines, known as CpG sites, although it has also been reported that DNA methylation can occur in other, non-CpG cytosines. Methylation at CpG sites located in the promoter region of a gene typically represses its expression (18). DNA methylation, one of the most studied epigenetic modifications involved in numerous biological processes (19), is associated with reduced gene expression, which are hypothesized to produce modification in child’s development, including an increased risk for the onset of psychopathology (20, 21). This process is potentially heritable but environmentally modifiable, especially by early stressful experiences (22, 23), such as childhood abuse/maltreatment (24), interparental conflict (25), low socioeconomic status (26), and parental psychopathological difficulties (27).

Dopamine is an important neurotransmitter playing a key role in many functions, including implicit and incentive learning, motivation (28), goal- and reward-seeking (29), aggression (30), and cognitive–behavioral processes functional in exploration (31), in novelty- and sensation-seeking (32) as well as in affiliative behaviors (33). Levels of this neurotransmitter depend on the activity of the dopamine transporter (DAT), which mediates the reuptake of released dopamine into the pre-synaptic terminal, thus terminating its action. It is very susceptible to epigenetic modifications (34). In humans, DAT1 has a polymorphic 40–base pair (bp) variable number of tandem repeats (VNTR) in the 3′-untranslated region (3′ VNTR). It can be repeated 3–11 times (33), but several studies have evidenced that the actually most frequent polymorphisms of SLC6A3 are 9- or 10-repeat (35). Although it plays a crucial role in the genesis and maintenance of emotional–behavioral difficulties, to date very few studies have focused on possible association between epigenetic status of the promoter, particularly the 5′-untranslated region (5′-UTR), of the dopamine transporter 1 gene (DAT1, also known as SLC6A3) and children psychopathological risk (36, 37). Our group has been one of the first in this regard (38). In this regard, we found that children with ADHD have significantly lower DAT methylation levels if compared to a healthy control group. The association between methylation level and the severity of symptomatology were also dependent on the genotype, with children carrying a 10/10 polymorphism showing greater psychopathological difficulties (38).

Furthermore, although several genetic studies have supported the growing evidence that the effects of familial environment on children’s emotional–behavioral functioning depend on the child’s genetic features (39–42), the findings are yet overall inconsistent. It is warranted to try improving the focus on epigenetic mechanisms, which could be responsible for mediating the effect of genotype × environment on children psychopathological difficulties (43, 44). As our previous study (38) has suggested, the association between methylation of DAT and children’s emotional–behavioral functioning was conditional on child’s vulnerability in their genotype (i.e., 10/10 polymorphism); however in that study, we did not take into account the role played by possible difficulties if not psychopathology in their parents. Actual child behavior may reflect the quality of interaction between paternal/maternal skills and individual ADHD temperament, in that severe ADHD may well incorporate a contribution by poor coping ability of the parents.

Based on the above premises and literature gap, the present study aimed to investigate the correlations and possible causal links between parental and offspring bio-genetics (presence of the 10-repeat allele in the 3′ VNTR region and methylation status of the 5′-UTR, both of DAT1 gene) and psychological–environmental factors (parental psychopathological symptoms and children’s emotional–behavioral functioning). In particular, we preliminary verified possible differences in methylation values between subjects with or without allele with 10 repeats in DAT1; then, given that correlations between psychological characteristics in parents and their children has been already ascertained by several studies in the Developmental Psychopathology framework (45–48), we explored: (1) correlations between biological, genetic, and psychological characteristics within the same individual (i.e., children, mothers, and fathers separately); (2) correlations between parental and offspring biological–genetic characteristics; (3) predictive effect of parental psychopathological symptoms on children’s biological–genetic characteristics; and (4) predictive effect of children’s biological–genetic characteristics on their own emotional–behavioral functioning.

Rooting on previous literature cited above, we hypothesized an association between biological, genetic, and psychological characteristics within the same individual and between parents and children. Moreover, we hypothesized a correlation between parental (especially maternal) biological–genetic characteristics and offspring psychopathological symptoms.

## Materials and Methods

### Sample Recruitment

Due to collaboration of public Primary Schools in the Center of Italy, we recruited 63 families with 6–10 years old children. In accordance with the Declaration of Helsinki, the study was approved before its start by the Ethical Committee of the Department of Dynamic and Clinical Psychology at Sapienza, University of Rome (protocol number 27/2016). Informed consent procedures included orally informing the children (using age-adequate approaches) and illustrating to parents the aims and scope of the study and all procedures and measures; the parents gave their written informed consent for their child to participate in this study. In addition, we declare that collected biological materials were used solely to the purpose of this study.

### Procedure for Biological Sampling

After receiving the consent of the primary school headmaster, a group of psychologists, specifically trained for the purposes of the study, reached children and parents at school premises. Participants (both parents and children) were assessed through buccal swabs (Isohelix Swab Pack). Buccal cell sampling is a feasible, non-invasive method that yields reproducible results also in DNA methylation studies (49). Subjects were aware of not having to eat (including chewing gum, candy, etc.), drink (except water), smoke, and brush their teeth for at least 1 h before sampling. Epithelial cell samples were carefully collected through the buccal swabs. The biological samplings were transported, slightly chilled by normal ice (+4°C), to the laboratories of the co-author, Esterina Pascale, for further processing.

After buccal swabs were gathered, mothers and fathers filled out independently self-report and report form questionnaires (described below). The order of administration of these measures was randomly selected. The following tools were chosen because they are very widely used and proved to be able to capture a wide range of difficulties that can be experienced by adults and children in general population (50, 51).

### Assessment of Parents’ Psychopathological Symptoms

Parents were administered the Symptom Check-List-90 item-Revised (SCL-90-R), a 90-item self-report questionnaire. It measures psychological symptoms and psychological distress in adult from general and clinical population (52). The SCL-90-R is rated on a Likert scale of 0 (not at all) to 4 (extremely) and asks participants to report if they have suffered in the past week from symptom such as: Headaches (*Somatization scale*), Trouble remembering things (*Obsessive-Compulsivity scale*), Feeling critical of others (*Interpersonal Sensitivity scale*), Blaming oneself for things (*Depression scale*), Feeling fearful (*Anxiety scale*), Feeling easily annoyed or irritated (*Hostility*), Feeling afraid to will faint in public (*Phobic Anxiety*), Feeling watched or talked about by others (*Paranoid Ideation scale*), and The idea that something is wrong with one’s mind (*Psychoticism scale*). Besides these nine primary scales, the questionnaire provides a Global Severity Index (GSI), used to determine severity and degree of psychological distress. The SCL-90-R showed good internal coherence (α = 0.72–0.96) in this study (Italian validated version, 51).

### Assessment of Children’s Emotional Behavioral Functioning

Parents also filled out the Italian version of the Child Behavior CheckList/6–18 (51, 53), which is one of the most widely used instruments to assess child and adolescent psychopathology both in epidemiological and clinical samples. The CBCL/6–18 is a 113-item informant-report questionnaire, which asks parents (independently) to rate specific emotional–behavioral problems of their child during the past 6 months. Items are rated on a 3-point Likert scale, ranging from “0” (not true) to “2” (very true or often true), and they are grouped into eight empirically based syndrome scales: anxious/depressed, withdrawn/depressed, somatic complaints, social problems, thought problems, attention problems, rule-breaking behavior, and aggressive behavior. These subscales are, in turn, combine in three broad-band scales: internalizing problems scale is comprised of items from the anxious/depressed, withdrawn-depressed, and somatic complaints scores; externalizing problems combines rule-breaking and aggressive behavior. There also is a total problems score, which is comprised the scores of all the problem items. For this study, the *T* scores of the internalizing and externalizing problems, and a total problems *T* score from both mother and father reports were used (53). Mean test–retest reliabilities of *r* = 0.88 have been reported for the school-age forms (53). In this study, statistical analyses were performed on raw scores. Furthermore, as suggested by several studies (54–56), data were obtained by mothers and fathers (independently). In fact, international literature has emphasized that parents may be discordant in the observation of their children.

### DNA Isolation and Genotyping

Buccal cell DNA isolations were performed using the Buccal-Prep Plus DNA isolation kit (Isohelix) according to the manufacturer’s instructions. The yield of DNA is usually between 3 and 10 µg. The 3′-UTR repeated sequence of DAT was amplified by the polymerase chain reaction (PCR) as it has been described previously (38, 57).

### Analysis of DNA Methylation

**Figure 1:**
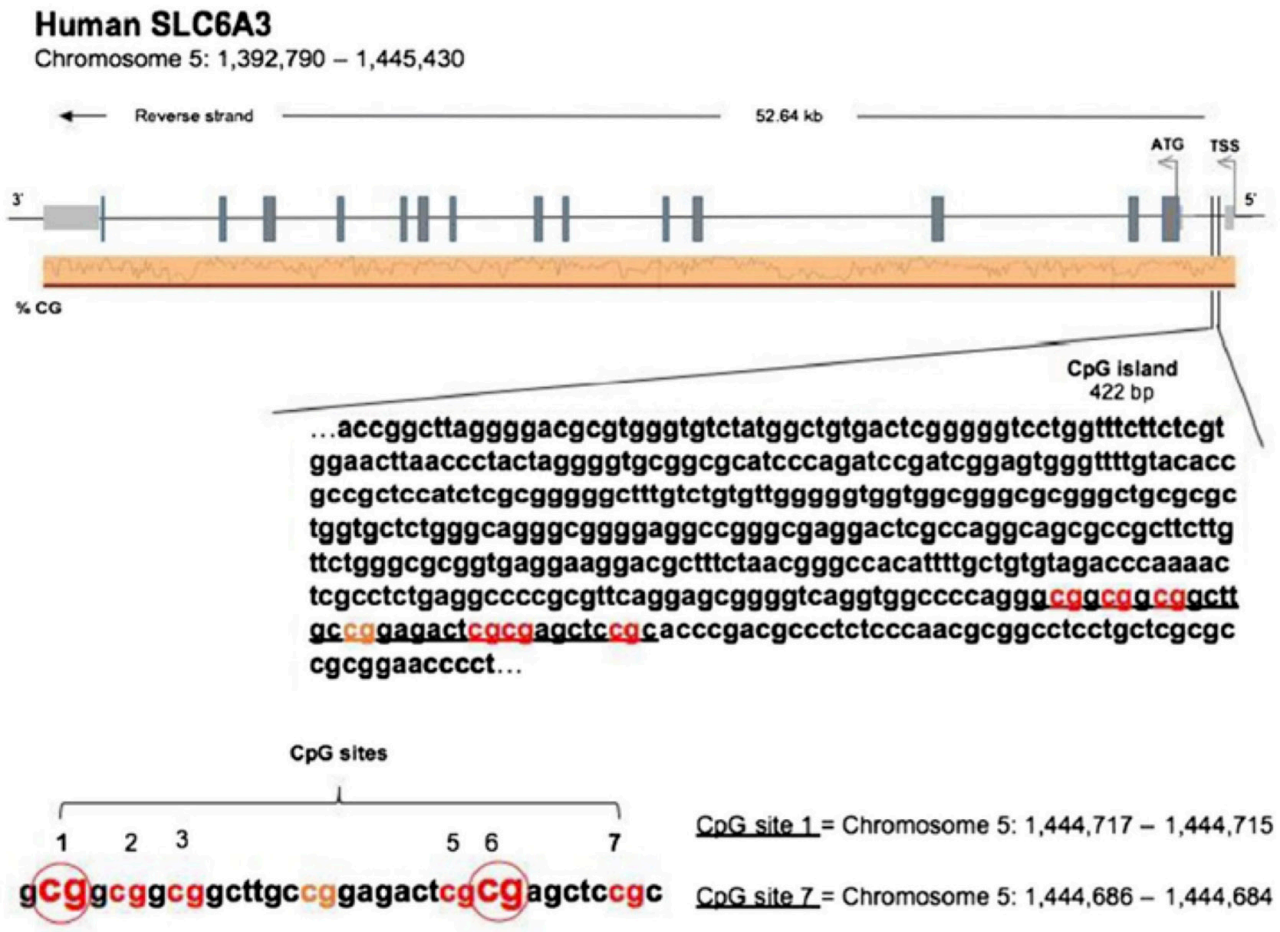
[Reproduced with permission of Springer, license no. 4198250303549, from Adriani et al. (38)]. Sequence of the 5′-untranslated region in the DAT gene, with localization of six chosen CpG residues. Our recent experimental work (58) discovered three out of six residues, which are relevant and useful for the purpose of association with the profile of ADHD symptoms’ severity. The same residues were used in this study.

DNAs from the buccal swabs were further processed for assessing amount of methylation in the DAT 5′-UTR sequence (notably, not the transcription promoter region). Amount of methylation was determined in six specific CpG residues [termed M1, M2, M3, M5, M6, and M7; see Figure 1, reproduced with permission of Springer, license n. 4198250303549, from Adriani et al. (38)]. Notably, M1–M3 represent a CGGCGGCGG motif, while M5/M6 represents a CGCG motif. The following primers (5′–3′) were used to amplify the gene for DAT: Fwd, AGCTACCATGCCCTA TGTGG; Rev, ATCAGCACTCCAAACCCAAC. Bisulfite-treated DNA was amplified by PyroMark PCR Kit (Qiagen, Hilden, Germany) in accordance with the manufacturer’s protocol. PCR conditions were as follows: 95°C for 15 min, followed by 45 cycles of 94°C for 30 s, 56°C for 30 s, 72°C for 30 s, and, finally, 72°C for 10 min. PCR products were verified by agarose electrophoresis. Pyrosequencing methylation analysis was conducted using the PyroMark Q24 (Qiagen, Hilden, Germany). The level of methylation was analyzed using the PyroMark Q24 Software (Qiagen, Hilden, Germany), which calculates the methylation percentage [mC/(mC + C)] for each CpG site, allowing quantitative comparisons (mC is methylated cytosine and C is unmethylated cytosine).

### Statistical Analysis

A preliminary screening of data showed few data missing for each psychometric instrument (3% for each instrument). Missing data were corrected using multiple imputation in SPSS software (version 23.0). To verify the possible differences in the state of methylation in subjects with 10-repeat allele polymorphism, preliminary descriptive analyses and a *T*-test for independent samples were carried out. Furthermore, bivariate Pearson correlations were conducted to verify aims 1 and 2 and regression analysis were conducted to verify aims 3 and 4 (see above in this manuscript). Also, we tested for the impact of confounding variables, such as smoking, alcohol use, concurrent medical illness, and traumatic experiences. All presented results were adjusted for these confounding variables.

## Results

### Sample Characteristics

For the aims of this study, the following cases were excluded from the sample: families with children with mental and/or physical disability (*N* = 4); families in which one or both parents could not understand the Italian language (*N* = 9); families who did not complete all the psychometric tools (*N* = 7); families in which one or more members were following a psychoactive pharmacological or psychological treatment (*N* = 10); families in which parents were not the biological parents of the child (*N* = 3); and families who refused to participate in the study (*N* = 9). Participants included 21 children (12 females and 9 males with age ranging from 6 to 10 years; average age 7.43 years and SD, 1.53), their mothers (average age 42.33 years and SD 5.21), and fathers (average age 44.91 years and SD 5.26). Most of the families recruited for the study (85.7%) had a middle socioeconomic status (59), and a large majority (95.2%) comprised intact family groups. Ninety percent of the families were Caucasian, and 80.9% relied on more than one income. Confounding variables (such as alcohol use, smoking, drugs of abuse, current medical illness, traumatic experiences, and social-economic status) were assessed through an anamnestic questionnaire, specifically created for this study.

**Figure 2:**
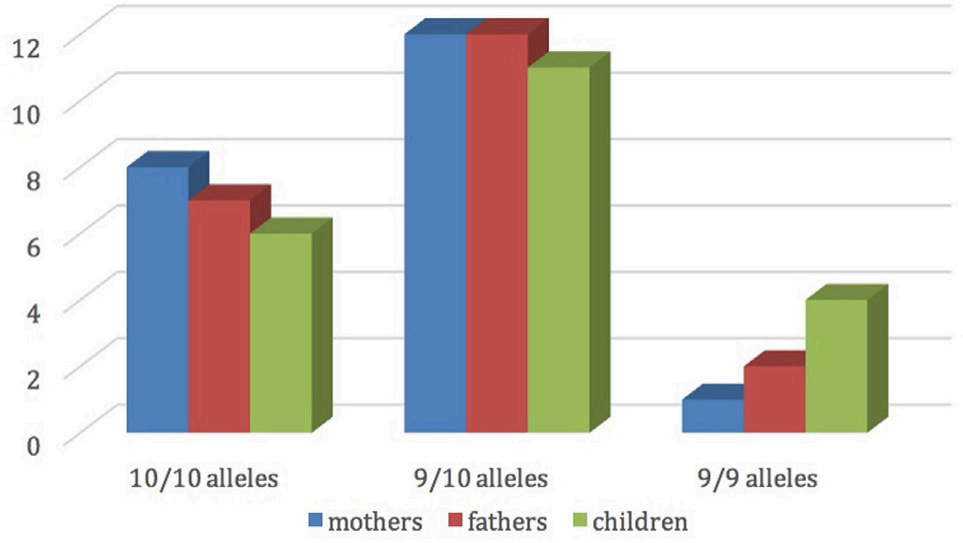
Mothers’, fathers’, and children’s dopamine transporter gene (DAT1).

Figure 2 shows the distribution of dopamine transporter gene among mothers, fathers and children.

Furthermore, Table 1 shows average scores and SDs of mothers and fathers’ psychopathological risk of parents, according to the subscales of the SCL-90-R, and the scores of the children’s emotional–behavioral functioning at questionnaires filled out by mothers and fathers.

**Table 1:** Average scores and SDs of mothers’ and fathers’ scores on SCL-90-R and CBCL-16/18.

	Mothers	Fathers
Somatization	0.45 (0.45)	0.24 (0.29)
Obsessive-compulsivity	0.44 (0.56)	0.29 (0.44)
Interpersonal sensitivity	0.24 (0.39)	0.21 (0.35)
Depression	0.28 (0.28)	0.2 (0.29)
Anxiety	0.3 (0.36)	0.2 (0.3)
Hostility	0.19 (0.23)	0.24 (0.42)
Phobic anxiety	0.05 (0.13)	0.01 (0.06)
Paranoid ideation	0.33 (0.39)	0.31 (0.42)
Psychoticism	0.13 (0.23)	0.09
Global Severity Index	0.27 (0.32)	0.2 (0.26)
CBCL internalizing problems	50.62 (9.53)	48.38 (8.88)
CBCL externalizing problems	46.95 (9.97)	45.24 (8.57)
CBCL total problems	47.9 (8.54)	46.86 (9.91)

### Differences in Methylation Values between Subjects with or without DAT1 10-Repeat Allele

Preliminarily, we wanted to verify possible differences in the state of methylation of DAT1 based on the presence of the 10-repeat allele on DAT1 gene; thus, an independent samples *T* test has been conducted. Independent variables were a two-level genotype groups, based on the number of 10-repeat allele copies (group 9/10, with one allele “10” and group 10/10, with two alleles “10”), while dependent variables were the levels of methylation of DAT1 5′-UTR at all considered sites [M1, M2, M3, M5, M6, and M7; see Ref. (38), for the rationale of this choice]. Results showed that there were no differences between groups on levels of methylation, neither in mothers (at all CpG sites *p* > 0.05), fathers (at all CpG sites *p* > 0.05), and child (at all CpG sites *p* > 0.05).

### Aim 1: Correlations between Biological and Psychological Characteristics within the Same Individual (Mothers, Fathers, and Children)

Bivariate Pearson correlation analyses were performed separately for mothers, fathers and children. In mothers and fathers, we verified the possible presence of correlations between the methylation levels at the six-selected CpG sites (of DAT1 5′-UTR) and psychopathological symptoms (correlations run between all SCL-90-R subscales and at all loci of DAT methylation, for mothers and fathers). Tables 2 and 3 shows significant correlations.

**Table 2:** Correlation between mothers’ CpG site methylation of DAT1 5′-untranslated region and their own psychopathological symptoms.

		CpG sites		
		M1	M6	M7
SCL-90-R	Anxiety	*r* = −0.14^a^	*r* = −0.48*	*r* = −0.21^a^
	Phobic anxiety	*r* = −0.45*	*r* = −0.49*	*r* = −0.53*

*p < 0.05.

*^a^Not significant (Ns)*.

**Table 3:** Correlation between fathers’ CpG site methylation of DAT1 5′-untranslated region and their own psychopathological symptoms.

		CpG site methylation
		M6
SCL-90-R	Depression	*r* = −0.56**
	Anxiety	*r* = −0.50*
	Hostility	*r* = −0.45*
	Psychoticism	*r* = −0.52*
	Global Severity Index	*r* = −0.44*

*p < 0.05.

**p < 0.01.

For children, we verified the possible presence of correlations between the methylation levels at the six selected CpG sites (of DAT1 5′-UTR) and their own (parent-reported) emotional–behavioral functioning (correlations run between all CBCL/6–18 subscales, as reported by mothers and fathers, and at all children’s loci of DAT methylation). Table 4 shows significant correlations.

**Table 4:** Correlation between children’s CpG site methylation of DAT1 5′-untranslated region and their own emotional–behavioral functioning.

		CpG site methylation	
		M2	M6
CBCL/6–18_mothers	Somatic complaints	*r* = 0.48*	*r* = 0.15^a^
	Internalizing problems	*r* = 0.47*	*r* = 0.36^a^
	Withdraw	*r* = 0.33^a^	*r* = 0.46*
CBCL/6–18_fathers	Attention problems	*r* = 0.51*	*r* = 0.27^a^

*p < 0.05.

*^a^Ns*.

### Aim 2: Correlations between Parental and Offspring Biological Characteristics

**Figure 3:**
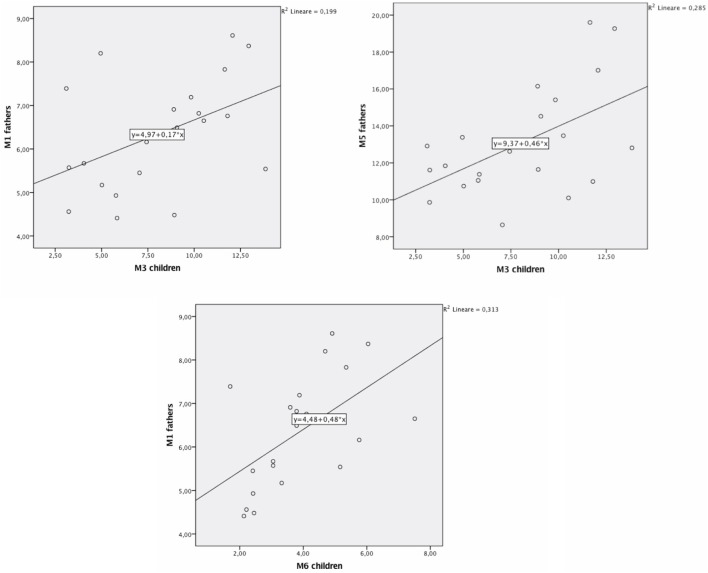
Scatter plots of correlations between fathers’ methylation vs. children’s methylation.

We carried out bivariate Pearson correlations to verify the presence of correlations between parents and children’s DAT methylation (at all considered CpG sites). Results showed no significant correlations between mothers and children’s DAT methylation. Instead, significant correlations were found between fathers’ methylation at CpG M1 and children’s methylation at CpG M3 (*r* = 0.45; *p* < 0.05; Figure 2), fathers’ methylation at M5 and children’s methylation at M3 (*r* = 0.53; *p* < 0.05; Figure 3), and fathers’ methylation at M1 and children’s methylation at M6 (*r* = 0.56; *p* < 0.01). Figure 3 shows scatter plots of correlations between fathers’ methylation vs. children’s methylation at different CpG positions.

### Aim 3: Predictive Effect of Parental Psychopathological Symptoms on Children’s Biological Characteristics

**Figure 4:**
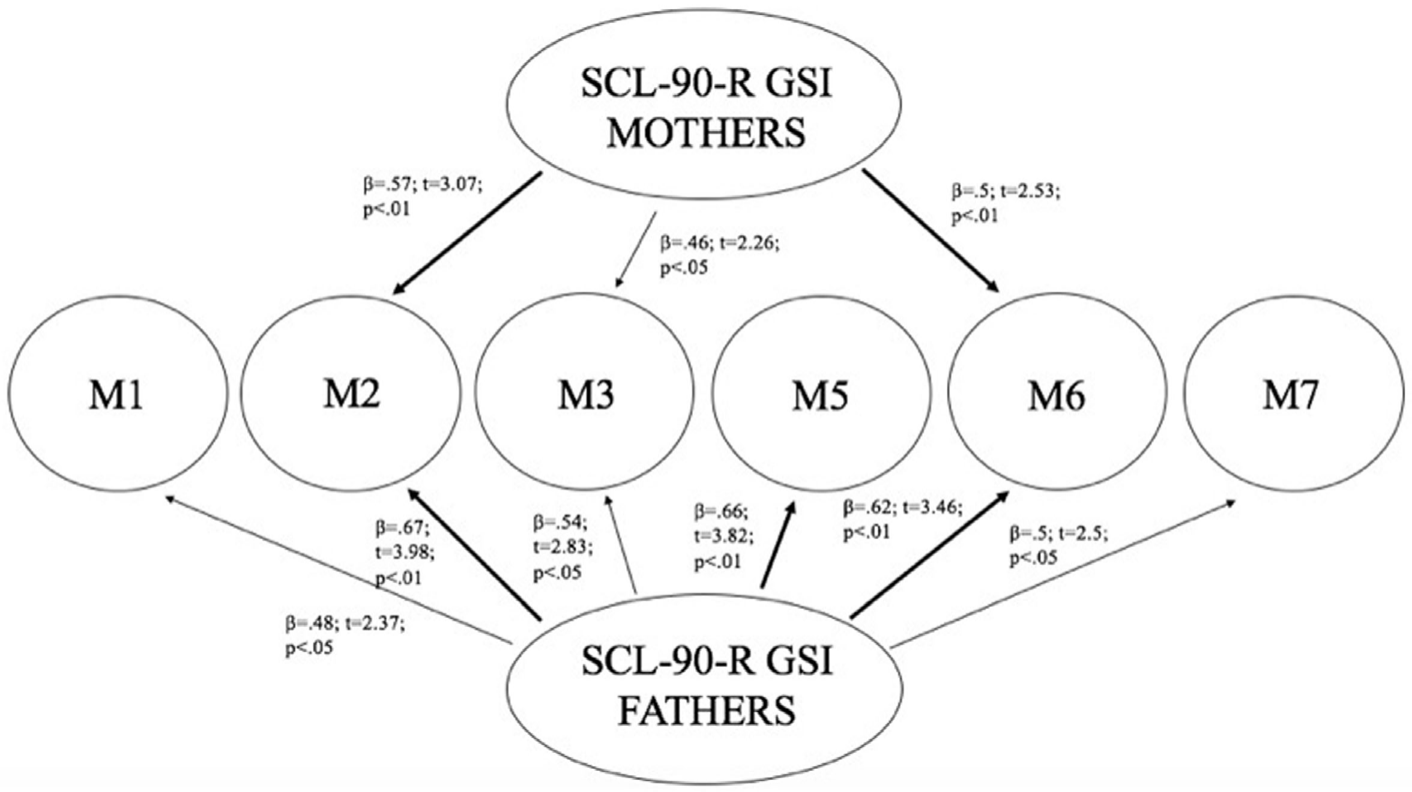
Predictive effect of parents’ psyche-pathological risk on children’s level of methylation, as a function of the specific CpG site at DAT1 5′-untranslated region.

Linear regression analysis has been performed using mothers and fathers’ SCL-90-R GSI as predictors, and children’s methylation of DAT (in all CpG sites) as dependent variables. Results showed that mothers’ GSI predicted children’s levels of methylation particularly at CpG M2 (*R*^2^ = 0.33; β = 0.57; *t* = 3.07; *p* < 0.01) and at CpG M6 (*R*^2^ = 0.25; β = 0.5; *t* = 2.53; *p* < 0.01), whereas its predictive power on methylation at CpG M3 was weaker (*R*^2^ = 0.21; β = 0.46; *t* = 2.26; *p* < 0.05). Furthermore, fathers’ GSI predicted children’s levels of methylation particularly at CpG sites: M2 (*R*^2^ = 0.45; β = 0.67; *t* = 3.98; *p* < 0.01), M5 (*R*^2^ = 0.43; β = 0.66; *t* = 3.82; *p* < 0.01), and M6 (*R*^2^ = 0.39; β = 0.62; *t* = 3.46; *p* < 0.01), whereas its predictive power on methylation at M1 (*R*^2^ = 0.23; β = 0.48; *t* = 2.37; *p* < 0.05), M3 (*R*^2^ = 0.3; β = 0.54; *t* = 2.82; *p* < 0.05), and M7 (*R*^2^ = 0.25; β = 0.5; *t* = 2.5; *p* < 0.05) was weaker. Figure 4 shows the relationships between the investigated variables, with the relative regression indices and arrows in bold indicating stronger predictive power.

### Aim 4: Predictive Effect of Children’s Biological Characteristics on Their Own Emotional–Behavioral Functioning

As Figure 4 shows, mothers and fathers’ psychopathological risk predicted children’s methylation of DAT at specific CpG sites. As seen in the previous analysis (see Table 4), methylation of DAT at CpG M2 and M6 loci were correlated with some of the subscales of CBCL 6–18 (somatic complains, internalizing problems, attention problems, withdraw), concerning the children themselves.

Therefore, we verified whether the children’s scores on these sub-dimensions were predicted by children’s methylation.

**Figure 5:**
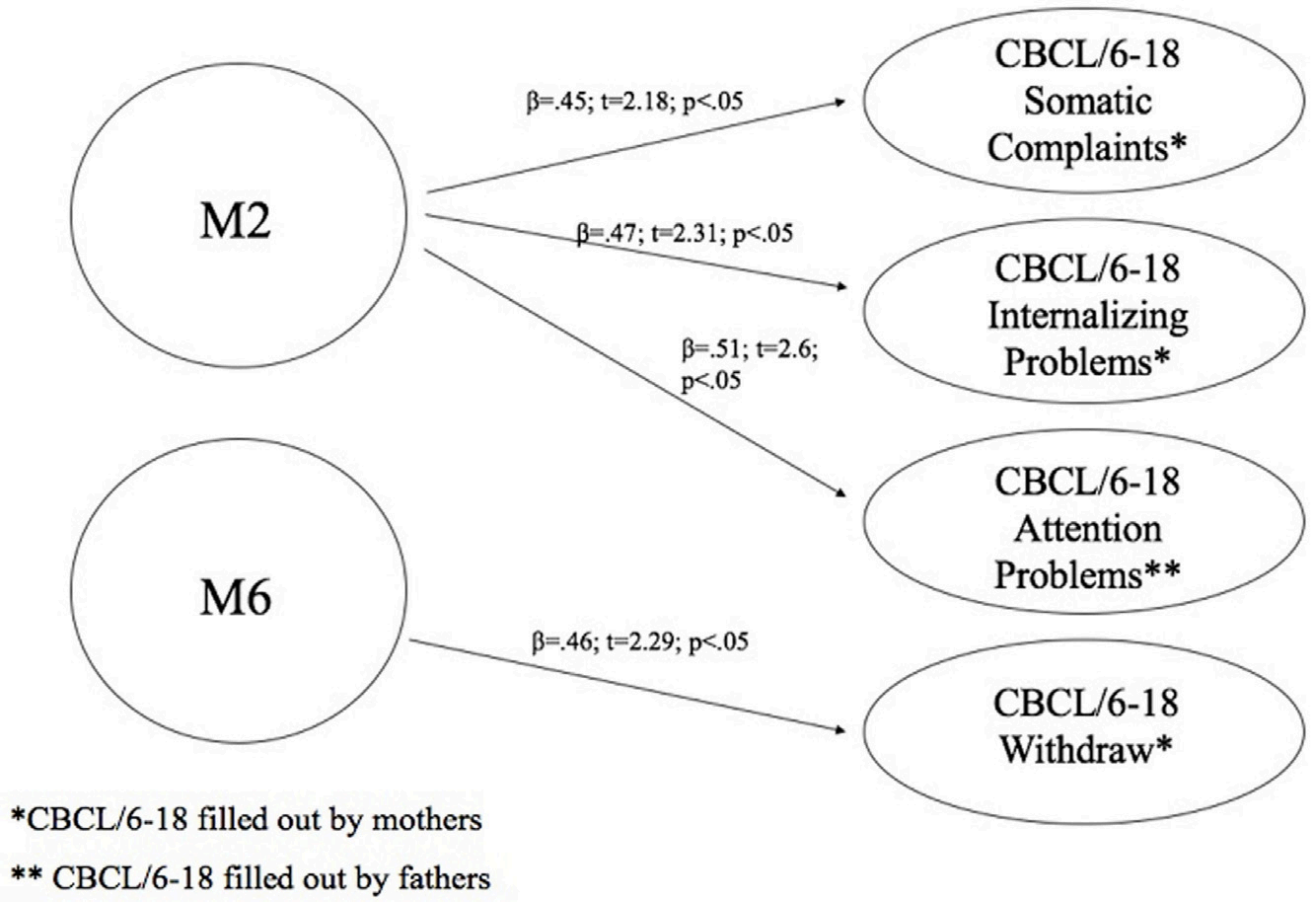
Predictive effect of children’s biological characteristics (CpG methylation at DAT1 5′-untranslated region) on their own emotional–behavioral functioning.

At this aim, we carried out linear regression analysis using methylation of DAT as predictors, while the subscales of CBCL/6–18 questionnaires compiled by mothers and fathers were used as dependent variables. Children’s methylation of DAT at CpG M2 predicted scores of somatic complaint (*R*^2^ = 0.2; β = 0.45; *t* = 2.18; *p* < 0.05) and internalizing problems reported by mothers (*R*^2^ = 0.22; β = 0.47; *t* = 2.31; *p* < 0.05), and attention problems reported by fathers (*R*^2^ = 0.26; β = 0.51; *t* = 2.6; *p* < 0.05). Furthermore, children’s methylation of DAT at CpG M6 predicted scores of withdraw reported by mothers (*R*^2^ = 0.22; β = 0.46; *t* = 2.29; *p* < 0.05). Figure 5 shows the relationships between the investigated variables, with the relative regression indices and arrows in bold indicating stronger predictive power.

## Discussion

The present study used a bio-psycho-social model, incorporating biological and genetic measures in the psychosocial–environmental perspective of Developmental Psychopathology, which widely demonstrated that negative developmental outcomes in children are linked to mothers and fathers’ psychopathological risk (60). Several authors in genetic and biological research have focused on the dopaminergic system (central and peripheral) as a crucial feature regulating emotional–behavioral functioning in children, as it is highly involved in attentional, motivational, and reward mechanisms (57, 61–63). These mechanisms regulate and shape interactional human characteristics and can modulate offspring capacity to cope with their parents’ psychological problems. Therefore, we chose to study DAT methylation and genotype, adding to previous literature by including fathers in our study and focusing on school-age children in a community sample.

Our previous study (38) and other authors (64) found that DAT methylation levels in offspring were correlated to their 10/10 3′-VNTR genotypes, in turn related to a more severe ADHD symptomatology as well as resistance to therapeutic intervention; therefore, we preliminarily explored again this possible link. However, this result was presently not confirmed in our sample. This incongruence can be due to many reasons: (1) the relative paucity of our sample, (2) the fact that children were observed at a single time-point, with no intervention intended to relief their situation, and/or (3) the fact that this study involved a general population, in which maladaptive bio-genetic-psychological characteristics are less marked than in clinical samples. Roth (20) demonstrated that, in general, DNA methylation alterations are complexly correlated with psychological and social–contextual factors, which frequently include suffering from psychopathological symptoms. Thus, our first aim was to explore possible correlations between DAT methylation and the presence of maladaptive behavior in the same individual: we focused on psychopathological symptoms in parents and on maladaptive emotional–behavioral functioning in children. In our study, DAT methylation at CpG sites M1, M6, and M7 in mothers was correlated with maternal Anxiety and Phobic Anxiety, whereas methylation in fathers at CpG M6 was related to paternal Depression, Anxiety, Hostility, Psychoticism and a higher score in GSI at SCL-90-R. In children, DAT methylation at CpG M2 was correlated with their somatic complaints, internalizing problems, and attention problems, whereas M6 was related to withdraw.

These results are noteworthy in that they confirm that subjects (both adults and offspring), when suffering from psychological difficulties, can show DAT methylation in several sites of the 5′-UTR promoter region, and that specificity seems to exist in mother’s, father’s and children’s bio-psychological profiles: mothers show DAT methylation in three CpG sites but the correlated symptoms are all in the area of anxiety; fathers show methylation in only one specific CpG, but their symptoms belong to a broader range of problems (e.g., depression, anxiety, and hostility); on the other hand, children show DAT methylation in two specific CpG sites and their symptoms are even broader than fathers’, also including Somatic Complaints and Attention Problems. Of course, these results must be confirmed by analyses on a wider sample, but we can still note a sort of pattern in the data: first of all, the CpG M6 is tapped in all these subjects; then, mothers’ altered dopaminergic system is seemingly impacting their capacity of coping with anxious symptoms, while fathers and children are facing a more general maladaptive psychological functioning. The interpretation of at least one result, however, is quite clear. Previous studies, in fact, have shown DAT methylation in children with ADHD from clinical samples (36, 37); in our study, we found attention problems in children of a general sample. It can be argued that these children share vulnerability for attention difficulties and behavioral disorganization, which in clinical samples are more severe, whereas in general population they reach sub-clinical levels.

We also explored possible correlations between DAT methylation in mothers, or fathers, and their children. Surprisingly, we found correlations only for the methylation at some very specific CpG sites in fathers and children. No methylated CpG in mothers correlated with any methylated sites in offspring. This result come as unexpected, as the vast majority of studies in this field found such correlations. Although it is intuitive and almost certainly true that the transmission of epigenetic and biological characteristics has a privileged path in the mother-infant *in utero* and peri-natal processes, it could still be possible that fathers have a much stronger weight in passing epigenetic and biological information through generations, associated with their lifestyle and due to yet unexplored biological and relational channels (65–67).

With the purpose of understanding the possible causal links between the parental psychopathological symptoms and, transgenerationally, DAT methylation in their children as well as, in turn, between the latter and their own emotional–behavioral functioning, we performed two subsequent linear regressions. The first, explored the possible links between the GSI scores of parents (separately for mothers and fathers) and DAT methylation in children: our results showed that GSI in mothers was associated with offspring DAT methylation at CpG sites M2, M3, and M6; paternal GSI was associated with offspring DAT methylation at all CpGs we investigated (M1, M2, M3, M5, M6, and M7). Consequently, we explored whether children’s DAT methylation could predict the characteristics of their own emotional–behavioral functioning. In fact, CpG M2 predicted specific offspring somatic complaints, attention problems, internalizing problems, whereas M6 predicted their withdraw.

DAT1 had been already associated with a wide range of psychopathologies in childhood, including ADHD (57, 63), conduct disorder (68), and oppositional defiant disorder (69). Moreover, in adult clinical samples, it has also been found associated with major depression (70), ADHD (71), post-traumatic stress disorder (72), obsessive–compulsive disorder (73), and alcoholism (74). Yet, despite these evidences, very few studies have focused on the specific role played by DAT in sub-clinical forms of psychopathological difficulties and in emotional–behavioral functioning of school-age children belonging to community samples (75). This is the first study to do so, to the best of our knowledge, which includes triplets of subject belonging to the same family (mothers, fathers, and children).

### Possible Limitations, Strength, and Implications

This study has some limitations. First, parental psychopathology and children’s emotional–behavioral functioning were assessed, respectively, through self-report and report form measures. Further studies should evaluate these variables through clinical observations and/or clinical interviews, to obtain more robust data on psychological profiles of the subjects (76, 77). Nonetheless, one merit of the present study was to consider the point of view of both mothers and fathers on the psychological functioning of their child, maintaining the differences that have been demonstrated in previous literature (54–56) and that concur to a more accurate representation of offspring mental health.

Second, our sample was relatively small. It must be stressed, however, that no other study has, to our knowledge, focused on parents and children in this developmental stage (school-age) by assessing both genotype and methylation, with a comparable attention to psychopathological symptoms. Although rarely performed, the study of psychopathological symptoms in community samples is supported by emerging literature underlining that up to a fifth of normative populations may show psychiatric problems (78). We acknowledge the fact that regression analyses suggesting a causal link between variables in cross-sectional studies cannot be considered conclusive in their results, due to limited effectiveness of this methodology outside longitudinal research. Nevertheless, some authors (79) have suggested the usefulness of cross-sectional studies even for assessing causal links between variables, and other authors have suggested that results from relatively small samples are still informative for the programming of assessment protocols and intervention plans (80), paving the way to more advanced investigations.

Our preliminary results suggest that epigenetic processes (such as DNA methylation) dynamically regulate our genomes responding to early environmental experiences (such as exposure to parental psychopathological symptoms in the first years of life), but they do so not just during the early stages of development but even in school-age children (81). Furthermore, we found that DNA methylation correlated with sub-clinical psychopathological symptoms, while previous literature focused only on psychiatric conditions, such as Mood disorders (82), Post-traumatic stress disorder (83), and Schizophrenia (84–86). Besides, this study may have important clinical implication for the prevention and treatment of emotional–behavioral difficulties in children, as it adds to previous knowledge about the role of genetic and environmental factors in predicting psychopathological symptoms within non-clinical populations. This approach can be useful for the identification of early indicators of maladaptive psychological functioning, observed in children well before they crystalize in adolescence and adulthood. Moreover, such studies (that assess individual genetic, biological, and psychological characteristics) can be essential for understanding the differential proneness of vulnerable versus resilient individuals for the onset of symptoms, secondary to inadequate environments and/or relationships (in this case, parental psychopathological symptoms). In fact, it has been demonstrated that children and parents with altered phenotype in dopaminergic system may be less emotionally attuned, due to a reduced sensitivity to reward and reinforcement (87).

## Ethics Statement

The study was carried out in accordance with the recommendations of the Declaration of Helsinki, before the start of the study, the Ethical Committee of the Department of Dynamic and Clinical Psychology at Sapienza, University of Rome approved it (protocol number 27/2016), and all subjects signed written informed consent.

## Author Contributions

SC contributed to the conception or design of the work, interpreted results, and drafted the introduction section. LC contributed to the conception or design of the work and drafted the discussion section. GB contributed to data collection and data analysis. EM contributed to data collection and data analysis. EP critically revised the article and interpreted results. CD critically revised the article and contributed to data analysis. WA contributed to the conception or design of the work and interpreted results. RT critically revised the article. All authors revised and approved the final version of the manuscript.

## Conflict of Interest Statement

There is only one item for potential conflict of interest to disclose: WA, Laviola G., EP, and CD—Metodo per determinare il deficit di attenzione con iperattività (method to determine attention deficit and hyperactivity disorder). Patent Application, deposit in: Italy at no. 102016000129938 (22 December 2016).

## References

[1] Dick DM. Gene-environment interaction in psychological traits and disorders. Annu Rev Clin Psychol (2011) 7:383–409. 10.1146/annurev-clinpsy-032210-104518 21219196 PMC3647367

[2] Davies PT Cicchetti D Toward an integration of family systems and developmental psychopathology approaches. Dev Psychopathol (2004) 16(3):477–81. 10.1017/S0954579404004626 15605621

[3] Cicchetti D Blender JA. A multiple-levels-of-analysis perspective on resilience: implications for the developing brain, neural plasticity, and preventive interventions. Ann N Y Acad Sci (2006) 1094:248–58. 10.1196/annals.1376.029 17347356

[4] Cicchetti D Curtis WJ Multilevel perspectives on pathways to resilient functioning. Dev Psychopathol (2007) 19(3):627–9. 10.1017/S0954579407000314 17972420

[5] Birnbaum LS Jung P. From endocrine disruptors to nanomaterials: advancing our understanding of environmental health to protect public health. Health Aff (2011) 30(5):814–22. 10.1377/hlthaff.2010.1225 21555467

[6] Hackman DA Farah MJ Meaney MJ. Socioeconomic status and the brain: mechanistic insights from human and animal research. Nat Rev Neurosci (2010) 11(9):651–9. 10.1038/nrn2897 20725096 PMC2950073

[7] Plass-Christl A Haller AC Otto C Barkmann C Wiegand-Grefe S Hölling H Parents with mental health problems and their children in a German population based sample: results of the BELLA study. PLoS One (2017) 12(7):e0180410 10.1371/journal.pone.0180410 28671981 PMC5495394

[8] van der Pol LD Groeneveld MG Endendijk JJ van Berkel SR Hallers-Haalboom ET Bakermans-Kranenburg MJ Associations between fathers’ and mothers’ psychopathology symptoms, parental emotion socialization, and preschoolers’ social-emotional development. J Child Fam Stud (2016) 25(11):3367–80. 10.1007/s10826-016-0490-x 27795659 PMC5061838

[9] Wlodarczyk O Pawils S Metzner F Kriston L Klasen F Ravens-Sieberer U. Risk and protective factors for mental health problems in preschool-aged children: cross-sectional results of the BELLA preschool study. Child Adolesc Psychiatry Ment Health (2017) 11(1):12. 10.1186/s13034-017-0149-4 28286550 PMC5341413

[10] Eaton NR Krueger RF Oltmanns TF. Aging and the structure and long-term stability of the internalizing spectrum of personality and psychopathology. Psychol Aging (2011) 26(4):987–93. 10.1037/a0024406 21728443 PMC3205340

[11] Hannigan LJ Walaker N Waszczuk MA McAdams TA Eley TC. Aetiological influences on stability and change in emotional and behavioural problems across development: a systematic review. Psychopathol Rev (2017) 4(1):52–108. 10.5127/pr.038315 28337341 PMC5360234

[12] Davidson RJ Lewis DA Alloy LB Amaral DG Bush G Cohen JD Neural and behavioral substrates of mood and mood regulation. Biol Psychiatry (2002) 52(6):478–502. 10.1016/s0006-3223(02)01458-0 12361665

[13] Clark WR Grunstein M Are We Hardwired? The Role of Genes in Human Behavior. Oxford: Oxford University Press (2004). 336 p.

[14] Forbes EE Brown SM Kimak M Ferrell RE Manuck SB Hariri AR. Genetic variation in components of dopamine neurotransmission impacts ventral striatal reactivity associated with impulsivity. Mol Psychiatry (2009) 14(1):60–70. 10.1038/sj.mp.4002086 17893706 PMC2668513

[15] Vucetic Z Totoki K Schoch H Whitaker KW Hill-Smith T Lucki I Early life protein restriction alters dopamine circuitry. Neuroscience (2010) 168(2):359–70. 10.1016/j.neuroscience.2010.04.010 20394806 PMC2873068

[16] Albert PR What is a functional genetic polymorphism? Defining classes of functionality. J Psychiatry Neurosci (2011) 36(6):363–5. 10.1503/jpn.110137 22011561 PMC3201989

[17] Breiling A Lyko F. Epigenetic regulatory functions of DNA modifications: 5-methylcytosine and beyond. Epigenetics Chromatin (2015) 8(1):24. 10.1186/s13072-015-0016-6 26195987 PMC4507326

[18] Smith ZD Meissner A DNA methylation: roles in mammalian development. Nat Rev Genet (2013) 14(3):204–20. 10.1038/nrg3354 23400093

[19] Bird A DNA methylation patterns and epigenetic memory. Genes Dev (2002) 16(1):6–21. 10.1101/gad.947102 11782440

[20] Roth TL. Epigenetic mechanisms in the development of behavior: advances, challenges, and future promises of a new field. Dev Psychopathol (2013) 25(4 0 2):1279–91. 10.1017/S0954579413000618 24342840 PMC4080409

[21] Tronick E Hunter RG Waddington, dynamic systems, and epigenetics. Front Behav Neurosci (2016) 10:107 10.3389/fnbeh.2016.00107 27375447 PMC4901045

[22] Weinhold B Epigenetics: the science of change. Environ Health Perspect (2006) 114(3):A160–7. 10.1289/ehp.114-a160 16507447 PMC1392256

[23] Szyf M The early-life social environment and DNA methylation. Clin Genet (2012) 81(4):341–9. 10.1111/j.1399-0004.2012.01843.x 22236068

[24] Suderman M Borghol N Pappas JJ Pereira SMP Pembrey M Hertzman C Childhood abuse is associated with methylation of multiple loci in adult DNA. BMC Med Genomics (2014) 7(1):13. 10.1186/1755-8794-7-13 24618023 PMC4007631

[25] Beach SR Whisman MA. Genetics and epigenetics in family context: introduction to the special section. J Fam Psychol (2013) 27(1):1–2. 10.1037/a0031484 23421828

[26] Tehranifar P Wu HC Fan X Flom JD Ferris JS Cho YH Early life socioeconomic factors and genomic DNA methylation in mid-life. Epigenetics (2013) 8(1):23–7. 10.4161/epi.22989 23196856 PMC3549876

[27] Bergink V Larsen JT Hillegers MHJ Dahl SK Stevens H Mortensen PB Childhood adverse life events and parental psychopathology as risk factors for bipolar disorder. Transl Psychiatry (2016) 6(10):e929 10.1038/tp.2016.201 27779625 PMC5290348

[28] Wise RA Dopamine, learning and motivation. Nat Rev Neurosci (2004) 5(6):483–94. 10.1038/nrn1406 15152198

[29] Arias-Carrión O Stamelou M Murillo-Rodríguez E Menéndez-González M Pöppel E. Dopaminergic reward system: a short integrative review. Int Arch Med (2010) 3(1):24. 10.1186/1755-7682-3-24 20925949 PMC2958859

[30] de Almeida RM Ferrari PF Parmigiani S Miczek KA. Escalated aggressive behavior: dopamine, serotonin and GABA. Eur J Pharmacol (2005) 526(1):51–64. 10.1016/j.ejphar.2005.10.004 16325649

[31] Frank MJ Doll BB Oas-Terpstra J Moreno F. Prefrontal and striatal dopaminergic genes predict individual differences in exploration and exploitation. Nat Neurosci (2009) 12(8):1062–8. 10.1038/nn.2342 19620978 PMC3062477

[32] Laviola G Macri` S Morley-Fletcher S Adriani W. Risk-taking behavior in adolescent mice: psychobiological determinants and early epigenetic influence. Neurosci Biobehav Rev (2003) 27(1–2):19–31. 10.1016/S0149-7634(03)00006-X 12732220

[33] Johnson ZV Young LJ. Neurobiological mechanisms of social attachment and pair bonding. Curr Opin Behav Sci (2015) 3:38–44. 10.1016/j.cobeha.2015.01.009 26146650 PMC4486624

[34] Shumay E Fowler JS Volkow ND. Genomic features of the human dopamine transporter gene and its potential epigenetic states: implications for phenotypic diversity. PLoS One (2010) 5(6):e11067. 10.1371/journal.pone.0011067 20548783 PMC2883569

[35] VanNess SH Owens MJ Kilts CD. The variable number of tandem repeats element in DAT1 regulates in vitro dopamine transporter density. BMC Genet (2005) 6:55. 10.1186/1471-2156-6-55 16309561 PMC1325255

[36] Xu Y Chen XT Luo M Tang Y Zhang G Wu D Multiple epigenetic factors predict the attention deficit/hyperactivity disorder among the Chinese Han children. J Psychiatr Res (2015) 64:40–50. 10.1016/j.jpsychires.2015.03.006 25840828

[37] Ding K Yang J Reynolds GP Chen B Shao J Liu R DAT1 methylation is associated with methylphenidate response on oppositional and hyperactive-impulsive symptoms in children and adolescents with ADHD. World J Biol Psychiatry (2017) 18:291–9. 10.1080/15622975.2016.1224928 27676100

[38] Adriani W Romano E Pucci M Pascale E Cerniglia L Cimino S Potential for diagnosis versus therapy monitoring of attention deficit hyperactivity disorder: a new epigenetic biomarker interacting with both genotype and auto-immunity. Eur Child Adolesc Psychiatry (2017) 8:1–12. 10.1007/s00787-017-1040-9 28822049

[39] Caspi A McClay J Moffitt TE Mill J Martin J Craig IW Role of genotype in the cycle of violence in maltreated children. Science (2002) 297(5582):851–4. 10.1126/science.1072290 12161658

[40] Rutter M Silberg J. Gene-environment interplay in relation to emotional and behavioral disturbance. Annu Rev Psychol (2002) 53:463–90. 10.1146/annurev.psych.53.100901.135223 11752493

[41] Brunner HG Nelen M Breakefield XO Ropers HH van Oost BA Abnormal behavior associated with a point mutation in the structural gene for monoamine oxidase A. Science (1993) 262(5133):578–80. 10.1126/science.8211186 8211186

[42] Bremne JD Vermetten E. Stress and development: behavioral and biological consequences. Dev Psychopathol (2001) 13(3):473–89. 10.1017/S0954579401003042 11523844

[43] Schuch V Utsumi DA Costa TVMM Kulikowski LD Muszkat M. Attention deficit hyperactivity disorder in the light of the epigenetic paradigm. Front Psychiatry (2015) 6:126. 10.3389/fpsyt.2015.00126 26441687 PMC4585002

[44] Perroud N Zewdie S Stenz L Adouan W Bavamian S Prada P Methylation of serotonin receptor 3A in ADHD, borderline personality, and bipolar disorders: link with severity of the disorders and childhood maltreatment. Depress Anxiety (2016) 33(1):45–55. 10.1002/da.22406 26350166

[45] Goodman S Gotlib I Risk for psychopathology in the children of depressed mothers: a developmental model for understanding the mechanisms of transmission. Psychol Rev (1999) 106(3):458–90. 10.1037/0033-295X.106.3.458 10467895

[46] Rutter M. Pathways from childhood to adult life. J Child Psychol Psychiatry (1989) 30(1):23–51. 10.1111/j.1469-7610.1989.tb00768.x 2647779

[47] Garber J Dodge KA Domains of emotion regulation. In: Garber J Dodge KA, editors. The Development of Emotion Regulation and Dysregulation. Cambridge: Cambridge University Press (2004). p. 3–11.

[48] Sroufe LA. The concept of development in developmental psychopathology. Child Dev Perspect (2009) 3(3):178–83. 10.1111/j.1750-8606.2009.00103.x 20161376 PMC2780349

[49] Torrone DZ Kuriakose JS Moors K Jiang H Niedzwiecki MM Perera FF Reproducibility and intraindividual variation over days in buccal cell DNA methylation of two asthma genes, interferon γ (IFNγ) and inducible nitric oxide synthase (iNOS). Clin Epigenetics (2012) 4(1):3. 10.1186/1868-7083-4-3 22414378 PMC3305380

[50] Prunas A Sarno I Preti E Madeddu F Perugini M. Psychometric properties of the Italian version of the SCL-90-R: a study on a large community sample. Eur Psychiatry (2012) 27(8):591–7. 10.1016/j.eurpsy.2010.12.006 21334861

[51] Frigerio A Cattaneo C Cataldo M Schiatti A Molteni M Battaglia M Behavioral and emotional problems among Italian children and adolescents aged 4 to 18 years as reported by parents and teachers. Eur J Psychol Assess (2004) 20(2):124–33. 10.1027/1015-5759.20.2.124

[52] Derogatis LR Symptom Checklist-90-R: Administration, Scoring and Procedures Manual. 3rd ed Minneapolis: National Computer Systems (1994).

[53] Achenbach TM Rescorla LA Manual for the ASEBA Adult Forms & Profiles. Burlington, VT, USA: Research Center for Children, Youth, & Families, University of Vermont (2003).

[54] Hay DF Pawlby S Sharp D Schmücker G Mills A Allen H Parents’ judgements about young children’s problems: why mothers and fathers might disagree yet still predict later outcomes. J Child Psychol Psychiatry (1999) 40(8):1249–58. 10.1111/1469-7610.00541 10604403

[55] Christensen A Margolin G Sulloway M Interparental agreement on child behavior problems. Psychol Assess (1992) 4(4):419–25. 10.1037/1040-3590.4.4.419

[56] Jansen M Bodden DHM Muris P van Doorn M Granic I. Measuring anxiety in children: the importance of separate mother and father reports. Child Youth Care Forum (2017) 46(5):643–59. 10.1007/s10566-017-9402-5 28989266 PMC5608774

[57] Giana G Romano E Porfirio MC D’Ambrosio R Giovinazzo S Troianiello M Detection of auto-antibodies to DAT in the serum: interactions with DAT genotype and psycho-stimulant therapy for ADHD. J Neuroimmunol (2015) 15(278):212–22. 10.1016/j.jneuroim.2014.11.008 25468771

[58] Vandenbergh DJ Persico AM Hawkins AL Griffin CA Li X Jabs EW Human dopamine transporter gene (DAT1) maps to chromosome 5p15. 3 and displays a VNTR. Genomics (1992) 14(4):1104–6. 10.1016/S0888-7543(05)80138-7 1478653

[59] Bornstein MH Bradley RH Socioeconomic Status, Parenting, and Child Development. London: Routledge (2014).

[60] Rutter M Moffitt TE Caspi A Gene–environment interplay and psychopathology: multiple varieties but real effects. J Child Psychol Psychiatry (2006) 47(3-4):226–61. 10.1111/j.1469-7610.2005.01557.x 16492258

[61] Robbins TW Everitt BJ Drug addiction: bad habits add up. Nature (1999) 398(6728):567–70. 10.1038/19208 10217139

[62] Faraone SV Bonvicini C Scassellati C Biomarkers in the diagnosis of ADHD–promising directions. Curr Psychiatry Rep (2014) 16(11):497 10.1007/s11920-014-0497-1 25298126

[63] Thissen AJ Bralten J Rommelse NN Arias-Vasquez A Greven CU Heslenfeld D The role of age in association analyses of ADHD and related neurocognitive functioning: a proof of concept for dopaminergic and serotonergic genes. Am J Med Genet B Neuropsychiatr Genet (2015) 168(6):471–9. 10.1002/ajmg.b.32290 25586935

[64] Auerbach JG Zilberman-Hayun Y Atzaba-Poria N Berger A The contribution of maternal ADHD symptomatology, maternal DAT1, and home atmosphere to child ADHD symptomatology at 7 years of age. J Abnorm Child Psychol (2017) 45(3):415–27. 10.1007/s10802-016-0230-0 27873141

[65] Strober M Peris T Steiger H. The plasticity of development: how knowledge of epigenetics may advance understanding of eating disorders. Int J Eat Disord (2014) 47(7):696–704. 10.1002/eat.22322 24976293

[66] Day J Savani S Krempley BD Nguyen M Kitlinska JB. Influence of paternal preconception exposures on their offspring: through epigenetics to phenotype. Am J Stem Cells (2016) 5(1):11–8. 27335698 PMC4913293

[67] Finegersh A Rompala GR Martin DI Homanics GE. Drinking beyond a lifetime: new and emerging insights into paternal alcohol exposure on subsequent generations. Alcohol (2015) 49(5):461–70. 10.1016/j.alcohol.2015.02.008 25887183 PMC4469624

[68] Lahey BB Rathouz PJ Lee SS Chronis-Tuscano A Pelham WE Waldman ID Interactions between early parenting and a polymorphism of the child’s dopamine transporter gene in predicting future child conduct disorder symptoms. J Abnorm Psychol (2012) 120(1):33–45. 10.1037/a0021133 PMC3058552 21171728

[69] Lee SS Lahey BB Waldman I Van Hulle CA Rathouz P Pelham WE Association of dopamine transporter genotype with disruptive behavior disorders in an eight-year longitudinal study of children and adolescents. Am J Med Genet B Neuropsychiatr Genet (2007) 144(3):310–7. 10.1002/ajmg.b.30447 17192955

[70] Camardese G Di Giuda D Di Nicola M Cocciolillo F Giordano A Janiri L Imaging studies on dopamine transporter and depression: a review of literature and suggestions for future research. J Psychiatr Res (2014) 51:7–18. 10.1016/j.jpsychires.2013.12.006 24433847

[71] Šerý O Paclt I Drtílková I Theiner P Kopečková M Zvolský P A 40-bp VNTR polymorphism in the 3′-untranslated region of DAT1/SLC6A3 is associated with ADHD but not with alcoholism. Behav Brain Funct (2015) 11(1):21 10.1186/s12993-015-0066-8 26058807 PMC4472402

[72] Li L Bao Y He S Wang G Guan Y Ma D The association between genetic variants in the dopaminergic system and posttraumatic stress disorder: a meta-analysis. Medicine (2016) 95(11):e3074. 10.1097/MD.0000000000003074 26986136 PMC4839917

[73] Zhang S Jiang W Tang X Xu Q. Association study of dopamine transporter gene (DAT1) variable tandem repeat sequence (VNTR) with obsessive-compulsive disorder in Chinese Han population. Int J Clin Exp Med (2015) 8(3):4606–10. 26064393 PMC4443227

[74] Van Der Zwaluw CS Engels RC Buitelaar J Verkes RJ Franke B Scholte RH. Polymorphisms in the dopamine transporter gene (SLC6A3/DAT1) and alcohol dependence in humans: a systematic review. Pharmacogenomics (2009) 10(5):853–66. 10.2217/pgs.09.24 19450132

[75] Hayden EP Hanna B Sheikh HI Laptook RS Kim J Singh SM Child dopamine active transporter 1 genotype and parenting: evidence for evocative gene–environment correlations. Dev Psychopathol (2013) 25(1):163–73. 10.1017/S0954579412000971 23398760 PMC5292820

[76] Tambelli R Cerniglia L Cimino S Ballarotto G Parent-infant interactions in families with woman diagnosed with postnatal depression: a longitudinal study on the effects of a psychodynamic treatment. Front Psychol (2015) 11(6):1210 10.3389/fpsyg.2015.01210 PMC4531209 26322008

[77] Cimino S Cerniglia L Porreca A Simonelli A Ronconi L Ballarotto G Mothers and fathers with binge eating disorder and their 18–36 months old children: a longitudinal study on parent–infant interactions and offspring’s emotional–behavioral profiles. Front Psychol (2016) 7:580 10.3389/fpsyg.2016.00580 27199815 PMC4843107

[78] Thurston IB Curley J Fields S Kamboukos D Rojas A Phares V How nonclinical are community samples? J Community Psychol (2008) 36(4):411–20. 10.1002/jcop.20223

[79] Green SB How many subjects does it take to do a regression analysis? Multivariate Behav Res (1991) 1(3):499–510. 10.1207/s15327906mbr2603_7 26776715

[80] Burdette WJ Gehan EA Planning and Analysis of Clinical Studies. Springfield: Thomas (1970).

[81] Cicchetti D Developmental Psychopathology, Developmental Neuroscience. New York: John Wiley & Sons (2016). 4656 p.

[82] Zill P Baghai TC Schüle C Born C Früstück C Büttner A DNA methylation analysis of the angiotensin converting enzyme (ACE) gene in major depression. PLoS One (2012) 7(7):e40479. 10.1371/journal.pone.0040479 22808171 PMC3396656

[83] Koenen KC Uddin M Chang SC Aiello AE Wildman DE Goldmann E SLC6A4 methylation modifies the effect of the number of traumatic events on risk for posttraumatic stress disorder. Depress Anxiety (2011) 28(8):639–47. 10.1002/da.20825 21608084 PMC3145829

[84] Connor CM Akbarian S DNA methylation changes in schizophrenia and bipolar disorder. Epigenetics (2008) 3(2):55–8. 10.4161/epi.3.2.5938 18398310

[85] Cerniglia L Cimino S Ballarotto G Casini E Ferrari A Carbone P Motor vehicle accidents and adolescents: an empirical study on their emotional and behavioral profiles, defense strategies and parental support. Transp Res Part F Traffic Psychol Behav (2015) 35:28–36. 10.1016/j.trf.2015.09.002

[86] Dunn E Soare T Simpkin A Suderman M Raffeld M Smith A Timing of exposure to adversity explains more variability in DNA methylation in late childhood than recency or accumulation of exposure. Biol Psychiatry (2017) 81(10):S366 10.1016/j.biopsych.2017.02.632

[87] Belden AC Irvin K Hajcak G Kappenman ES Kelly D Karlow S Neural correlates of reward processing in depressed and healthy preschool-age children. J Am Acad Child Adolesc Psychiatry (2016) 55(12):1081–9. 10.1016/j.jaac.2016.09.503 27871643 PMC5131532

